# FAS Prevention Strategies: Passive and Active Measures

**Published:** 1994

**Authors:** Janet R. Hankin

**Affiliations:** Janet R. Hankin, Ph.D., is an associate professor in the Department of Sociology and associate director for tracking at the Fetal Alcohol Research Center, Wayne State University, Detroit, Michigan

## Abstract

FAS prevention programs attempt to reduce the incidence of drinking during pregnancy by educating women about the risks of alcohol to their unborn babies. Alcoholic beverage warning labels reach a wide audience and have had some effect on curtailing such drinking. Community-based efforts, however, reach specific populations of women and their health care providers and have a more potent, local effect.

When a pregnant woman drinks heavily,^1^ she increases the risks to her fetus for physical abnormalities, mental retardation, and behavior and learning problems, collectively called fetal alcohol syndrome (FAS) or fetal alcohol effects (FAE). Severe effects of alcohol consumption can occur during the first trimester of pregnancy, before many women even realize that they are pregnant (see the article by Jacobson and Jacobson, pp. 30–36).

Debate continues as to whether the risk to the fetus is a function of the amount of alcohol the woman consumes throughout pregnancy or if it is a function of the woman’s blood alcohol level at a particular phase in fetal development (National Institute on Alcohol Abuse and Alcoholism [NIAAA] 1991). Some women may consume large amounts of alcohol during pregnancy without causing obvious harm to the fetus, whereas others may drink moderate amounts of alcohol and see severe effects. Given these uncertainties, the Surgeon General has advised all women to abstain from alcohol consumption during pregnancy (Public Health Service 1981). Even with this warning, however, some pregnant women still drink (an estimated 20 percent of pregnant women drank some amount of alcohol during pregnancy in 1988 [NIAAA 1993]); either they are unaware of the warning or they do not heed the advice.

Prevention efforts attempt to reduce the incidence of FAS and FAE by making women in their childbearing years aware of the hazards of drinking during pregnancy. Because abstaining from alcohol prior to and throughout pregnancy is the only way to guarantee the birth of a child free of FAS/FAE, prevention programs try to target women before they become pregnant. However, the programs also should target women who are drinking during pregnancy, to increase their likelihood of having healthy babies in the current as well as subsequent pregnancies.

This article examines FAS and FAE prevention efforts in the United States. One such measure, alcoholic beverage warning labels, reaches all drinkers but is a passive technique that relies on the drinker to read, understand, remember, and comply with the warning. Also at issue is whether the woman sees the label (some are hard to find) and whether someone else pours the drink from the bottle for the woman (e.g., a waiter in a bar).

Other measures, such as intensive prenatal clinic programs, training programs for health care providers and teachers, and community-based programs, reach a smaller audience but are more active in educating women and the people who give women advice about prenatal health. Because not every prevention program can be described in this article, several programs that illustrate the range of approaches currently available are discussed.

## The Alcoholic Beverage Warning Label

On November 18, 1989, Public Law 100–690 was implemented, requiring warning labels on all alcoholic beverage containers sold or distributed in the United States. The law was instituted as a prevention strategy aimed at warning the public about the risks related to consuming alcohol. One of the warnings on the label addresses pregnant women. It states: “(1) According to the Surgeon General, women should not drink alcoholic beverages during pregnancy because of the risk of birth defects.” Now that the warning label has been in place for several years, it is appropriate to evaluate its effectiveness. Specifically, has drinking during pregnancy decreased with the advent of warning labels? As there has been only one other study of the effect of the warning label on the behavior of pregnant women (an unpublished paper by Kaskutas and Graves), this discussion will focus on our study—the only study with pre- and post-test data on a large number of pregnant women.

### Detroit Study of Pregnant Women

Our ongoing study in Detroit examines the impact of alcoholic beverage warning labels on African-American women who are pregnant. The study focuses on two questions: Has awareness of the warning label increased over time, and has drinking by pregnant women decreased since the alcoholic beverage warning label law took effect? Given the characteristics of the sample (inner city, African-American, low income [most of the women are welfare recipients]), the study provides a stringent test for the effectiveness of the warning label because the women are unlikely to be reached by other public health prevention efforts. As 80 percent of the pregnancies in the study population are unplanned and the women do not begin prenatal care until the second trimester, the warning label reaches them before health care providers do.

**GOVERNMENT WARNING:** (1) ACCORDING TO THE SURGEON GENERAL, WOMEN SHOULD NOT DRINK ALCOHOLIC BEVERAGES DURING PREGNANCY BECAUSE OF THE RISK OF BIRTH DEFECTS. (2) CONSUMPTION OF ALCOHOLIC BEVERAGES IMPAIRS YOUR ABILITY TO DRIVE A CAR OR OPERATE MACHINERY AND MAY CAUSE HEALTH PROBLEMS.

The sample consists of 3,572 inner-city women who initiated prenatal care at Hutzel Hospital Prenatal Clinic between June 1, 1989 (5 months before the warning label law took effect) and September 30, 1991. The average woman seeking care was 24 years old; had been pregnant twice before; and began prenatal care late, at 23 weeks gestational age (second trimester). To avoid researcher bias, we are kept unaware of the health of the babies.

Data were collected from each woman during her first prenatal clinic visit on (1) reported alcohol consumption around the time of conception and during the 2 weeks prior to her prenatal visit; (2) awareness of the alcoholic beverage warning label, which was measured by the question, “Is there a warning label on alcoholic beverages (i.e., something that says that alcohol may affect your health)?”; and (3) maternal age, gestational age of the fetus, and gravidity (number of pregnancies, including current one). (For details on the method of data collection, see Hankin et al. 1993*a*).

An informal survey of alcoholic beverage retailers collected information on how rapidly warning labels appeared on alcoholic beverages. Seven months after the law took effect (May–June 1990), bottles without warning labels were still on the shelves. Fourteen percent of beer, 31 percent of wine coolers, 66 percent of wine, and 70 percent of liquor containers still lacked warnings, which suggests that containers with longer shelf lives (wine and liquor) were less likely to carry warning labels (Hankin et al. 1993*b*).

#### Has Awareness of the Warning Label Increased?

**Figure 1:**
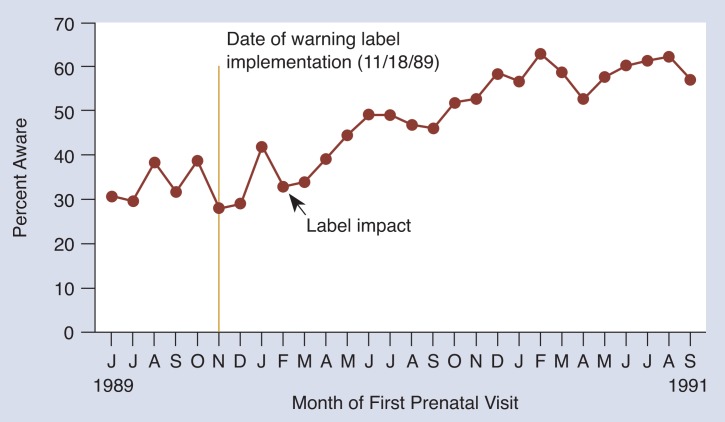
Awareness of the alcoholic beverage warning label among pregnant women in Detroit. “Label impact” represents the month when awareness of the label significantly increased.

Figure 1 shows that for the month of June 1989—5 months *before* implementation of the law—approximately 31 percent of the women indicated that they saw the warning label. This demonstrates a high rate of false positives (women who said incorrectly that they saw the warning label). The relatively high rate of false positives is consistent with other studies of warning label awareness (Hilton 1993). The high rate of false positives may represent acquiescence bias (i.e., the interviewer would not ask the question unless it was true), generalization of other warning messages to alcohol (i.e., cigarettes have warning labels, so alcohol must have warning labels), or awareness of the sulfite warning on some wine bottles (Hankin et al. 1993*b*). Almost 2 years after label implementation and more than 1 year after most bottles in stores contained labels, 57 percent of the women reported seeing the warning label on alcoholic beverages. Therefore, after some time lag, awareness of the label increased during the postlabel period.

#### Has Drinking Decreased?

The second question in the study addresses whether awareness of the warning label resulted in decreased drinking by pregnant women. This is not easy to determine because other factors, such as maternal age and number of previous pregnancies, may affect drinking trends over time and therefore obscure any changes that could be attributed to the warning label. For example, women who are older and have had multiple children tend to drink more than younger women who have not had children (Hankin et al. 1993*a*).

To address this problem, we calculated a drinking score for each woman that was adjusted for maternal age, weeks of gestation, and number of pregnancies. From the interview, we knew reported drinking at conception (prior to pregnancy recognition) and at the time of the first prenatal visit (after pregnancy recognition), two measures that could be compared to evaluate an effect of the warning label. As most women automatically tend to decrease their drinking during pregnancy (the alcohol does not taste good or has a different effect), to determine any effect of the warning label, drinking level at the time of the first prenatal visit also was adjusted for an expected decrease in drinking. A negative drinking score suggests that women have decreased their drinking after pregnancy recognition more than we expected them to. A continuation of the negative drinking scores after November 1989 suggests that implementation of the warning label has decreased the level of drinking during pregnancy.

For each month of the study, the drinking scores for women initiating prenatal care during that month were averaged. For example, the scores for women who began in June 1990 were averaged, and this number represents the value on the graph for that month. Separate analyses were completed for heavier (risk) and lighter (nonrisk) drinkers because it was hypothesized that the warning label would have a different impact on the two types of drinkers. For example, risk drinkers may be more likely to see the warning label because they drink more, but that may not translate into decreased drinking.

#### Nonrisk Drinkers

**Figure 2:**
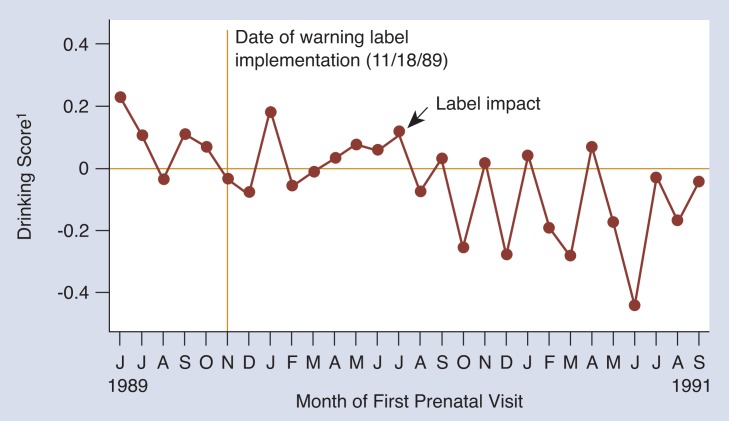
Impact of the warning label on the drinking of pregnant women who drink at nonrisk levels (consuming less than 0.5 ounces of alcohol per day). “Label impact” represents the month when drinking levels for these women began to decline in response to the warning label. ^1^Drinking scores were calculated for each woman using a mathematically complex set of analyses (see Hankin et al. 1993*c*). For each month, the scores for the women initiating care during that month were averaged and plotted on this graph. A negative drinking score suggests that women are drinking less than expected and that this decline is related to implementation of the warning label (see article for more detailed explanation).

Time series^2^ techniques were used to analyze the trends shown in figure 2 (see Hankin et al. 1993*c*). The results confirm that nonrisk drinkers decreased their drinking after the warning label was implemented. As predicted, there was a time lag between when the warning label was implemented and when it made an impact. A downward trend in nonrisk drinking did not appear until July 1990, 8 months after the warning label was implemented. Although nonrisk drinkers reduced the amount of alcohol they consumed, the decrease was small. It is estimated that nonrisk drinkers reduced their drinking by 0.05 ounces of absolute alcohol per week, or the equivalent of 1 ounce of beer (see Hankin et al. 1993*a*).

#### Risk Drinkers

**Figure 3:**
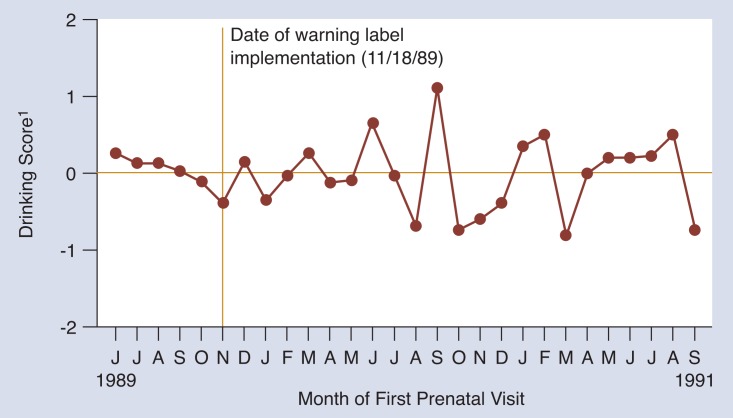
Impact of the warning label on the drinking of pregnant women who drink at risk levels (consuming 0.5 ounces or more of alcohol per day). This graph shows that the label had no statistically significant impact on drinking during pregnancy for these women. ^1^Drinking scores were calculated for each woman using a mathematically complex set of analyses (see Hankin et al. 1993*c*). For each month, the scores for the women initiating care during that month were averaged and plotted on this graph. A negative drinking score suggests that women are drinking less than expected and that this decline is related to implementation of the warning label (see article for more detailed explanation).

As figure 3 indicates, women who needed the warning the most, those drinking at risk levels, did not change the amount of alcohol they consumed after the warning label was implemented. This result is consistent with the findings reported by Andrews and colleagues (1991): risk drinkers were less likely to respond to the warning label.

Why do some women appear to ignore the warning label? One explanation is that 71 percent of the risk drinkers had been pregnant before. Although we were blinded to the outcome of the women’s births, we hypothesize that the women believe (rightly or wrongly) that their babies are healthy. Women in the study were asked how often it was safe to drink while pregnant and how likely it was that their baby would be healthy if they drank alcohol at risk levels. Responses suggest that pregnant women who previously gave birth to babies are more likely to feel safe drinking daily and to believe their future babies will be healthy if they drink heavily during pregnancy than do women who never gave birth. The consistency between the attitudes and behaviors of the women who previously had babies suggests a sense of personal invulnerability.

## Looking Beyond the Label

Although the study of pregnant women in Detroit demonstrated that the alcoholic beverage warning label resulted in a small decrease in drinking (0.05 ounces of absolute alcohol per week among nonrisk drinkers), the warning label does not appear to influence women drinking at risky levels. Other, more active prevention strategies, such as prenatal clinic programs, training programs for professionals, and community-based programs, have been developed to target individual women and their health care providers.

### Prenatal Clinic Programs

Prenatal clinic programs developed in Boston and Detroit are using counseling and intensive education approaches to reduce the incidence of FAS and FAE.

Beginning in the late 1970’s, Boston City Hospital developed the Fetal Alcohol Education Program, a voluntary program for reducing drinking among pregnant women.^3^ Women were offered counseling about drinking during pregnancy at their routine prenatal visits. The program avoided direct criticism in an effort to support rather than chastise expectant mothers (Rosett et al. 1981). It stressed abstinence from alcohol and emphasized that women increased their chances of having healthy babies if they stopped drinking. Attempts were made to increase the women’s self-esteem, and referrals were provided to specialized treatment programs and community agencies.

Rosett and colleagues (1983) studied the success of 49 risk drinkers who participated in the program. They reported that as a result of the program, 33 of the women abstained or significantly reduced their drinking by the third trimester of pregnancy.

An intensive FAS/FAE prevention program was designed at Wayne State University, in Detroit, in June 1993 to reduce drinking among women who drink at risky levels. This program, known as the Protecting the Next Pregnancy Project, is one of the components of Wayne State University’s Fetal Alcohol Research Center. The program is directed at inner-city women who have recently delivered babies exposed to risk levels of alcohol in utero. A concurrent study was set up to evaluate the effectiveness of the program. To be eligible for the study, a woman must have been interviewed in the prenatal clinic while seeking care for her pregnancy, and she must have admitted to drinking at least 0.3 ounces of absolute alcohol per day at the time of conceiving her baby. In addition, the baby must have been recently delivered at Hutzel Hospital, be at least 32 weeks gestational age, and weigh 1,500 grams or more. Other than birth weight, the researchers are blinded to the health status of the babies.

If a woman agrees to participate in the study, she is randomly assigned to either the experimental group or the control group. If she is assigned to the experimental group, she receives intensive counseling about moderating her drinking, with the goal being set of abstaining during her next pregnancy. The counseling is based on a cognitive-behavioral approach, stressing goal setting, keeping a diary, and coping techniques. If she is assigned to the control group, she receives a generalized program in health education with no drinking counseling.

The study will follow these women until they conceive and deliver another child. The hope is that women in the experimental group will reduce their drinking and that babies from future pregnancies will be healthier than the babies who were exposed to alcohol in utero. Because this study began in June 1993, none of the women has had a second child, and it is still too early to evaluate whether the goals have been achieved.

### Training Health Care Providers and Teachers

Between 1979 and 1981, 6,300 health care providers (e.g., physicians, nurses, alcohol counselors, social workers, psychologists) and teachers (those that may encounter pregnant women or their children) in King County, WA, were educated about the risks of drinking during pregnancy through the Pregnancy and Health Program. A variety of forums were used, including hospital lectures for physicians and other health care providers, continuing education courses, publications, and presentations at professional meetings. After the educational campaign was completed, all the participants reported that they knew more about FAS/FAE and were more likely to recommend that women abstain from alcohol use during pregnancy. An evaluation of the program revealed a significant increase in the number of physicians asking their patients about their alcohol consumption (NIAAA 1987).

### Community Prevention Efforts

In addition to training providers and teachers, King County, WA, launched a public health campaign that provided information about alcohol and pregnancy to 1 in 44 pregnant women in the Seattle area between 1974 and 1981. As a result, awareness and knowledge increased among these women regarding the risks of drinking during pregnancy (NIAAA 1987).

Another example of a successful community-based program is the Tuba City, AZ, Fetal Alcohol Syndrome Prevention Project, which focuses on preventing FAS among Navajo and Hopi Indians in Arizona (Masis and May 1991). The project operates prevention programs that involve communitywide FAS education, prenatal clinic screening for alcohol use, and education for women in prenatal clinics. For women identified as high risk because they drink heavily during their pregnancies and previously gave birth to a child with FAS or FAE, the prevention program includes detoxification, individual and group therapy, and voluntary birth control or sterilization services.

During the course of a study conducted by Masis and May (1991), 29 high-risk women were referred to the program during pregnancy. Of these pregnant women, 21 were seen before the third trimester. According to the researchers, 18 of these women reported abstaining from alcohol during their third trimester of pregnancy. Abstention during the third trimester improves birth weight of the infants and may have other beneficial effects. The researchers attribute the success of the program to a family-oriented approach and to the participation of tribal community leaders in the prevention efforts.

## Conclusions

The alcoholic beverage warning label has resulted in a modest effect on drinking by pregnant women, but more intensive efforts are necessary to reach women who drink heavily. As Weiner and colleagues (1989) suggest, there is a need to go beyond educational campaigns such as the warning label. They argue for programs tailored to intervene directly with pregnant women who are risk drinkers. For example, Masis and May (1991) promote a multifaceted approach that includes “outreach, case finding,^4^ and some community awareness” (p. 489).

Only a handful of FAS programs are described in this article. These efforts, and others like them, hold the promise for reducing the incidence of heavy drinking during pregnancy and improving birth outcomes. When it comes to stopping FAS, prevention is key.

## Footnotes

1 In this article, heavier (risk) drinking is defined as consuming at least 0.5 ounces or more of absolute alcohol per day (a little less than one mixed drink). Lighter (nonrisk) drinking is defined as consuming less than 0.5 ounces of absolute alcohol per day.

2 Time series analysis is a conservative statistical technique that allows researchers to detect upward or downward trends as well as seasonal patterns (see Hankin et al. 1993*b* for details).

3 This program is still active, but its focus has shifted toward health professionals rather than individual women. Also, see the article by Weiner and Morse, pp. 67–72.

4 “Case finding” in this context refers to such efforts as locating women who are drinking heavily or those who have previously delivered a child with FAS, and identifying women who plan to conceive.
